# Bidirectional transport of 2-chloroadenosine by equilibrative nucleoside transporter 4 (hENT4): Evidence for allosteric kinetics at acidic pH

**DOI:** 10.1038/s41598-019-49929-w

**Published:** 2019-09-19

**Authors:** David Tandio, Gonzalo Vilas, James R. Hammond

**Affiliations:** Department of Pharmacology, University of Alberta, Edmonton, Alberta Canada

**Keywords:** Carrier proteins, Ischaemia

## Abstract

Adenosine has been reported to be transported by equilibrative nucleoside transporter 4 (ENT4), encoded by the *SLC29A4* gene, in an acidic pH-dependent manner. This makes hENT4 of interest as a therapeutic target in acidic pathologies where adenosine is protective (e.g. vascular ischaemia). We examined the pH-sensitivity of nucleoside influx and efflux by hENT4 using a recombinant transfection model that lacks the confounding influences of other nucleoside transporters (PK15-NTD). We established that [^3^H]2-chloroadenosine, which is resistant to metabolism by adenosine deaminase, is a substrate for hENT4. Transport of [^3^H]2-chloroadenosine at a pH of 6.0 in PK15-NTD cells stably transfected with *SLC29A4* was biphasic, with a low capacity (V_max_ ~ 30 pmol/mg/min) high-affinity component (K_m_ ~ 50 µM) apparent at low substrate concentrations, which shifted to a high capacity (V_max_ ~ 500 pmol/mg/min) low affinity system (K_m_ > 600 µM) displaying positive cooperativity at concentrations above 200 µM. Only the low affinity component was observed at a neutral pH of 7.5 (K_m_ ~ 2 mM). Efflux of [^3^H]2-chloroadenosine from these cells was also enhanced by more than 4-fold at an acidic pH. Enhanced influx and efflux of nucleosides by hENT4 under acidic conditions supports its potential as a therapeutic target in pathologies such as ischaemia-reperfusion injury.

## Introduction

Equilibrative nucleoside transporter 4 (ENT4) is a member of the SLC29A family of solute transporters. It was initially identified as the plasma membrane monoamine transporter (PMAT), prior to its molecular identification as *SLC29A4*^1^. At neutral pH, hENT4 transports predominantly monoamines such as serotonin^2^. It was subsequently determined that hENT4 could also transport adenosine, but reportedly only at acidic pH^3^. Adenosine is well-established as an endogenous cardioprotective agent and vasodilator that attenuates tissue injury during an ischaemic episode^4,5^. Furthermore, intracellular acidification due to anaerobic glycolysis followed by extracellular acidification of tissues is a hallmark of ischaemia and early reperfusion^6,7^. This raises the possibility that hENT4 may be a target for drug development for pathological conditions associated with an acidic environment where adenosine is protective, such as in ischaemia-reperfusion injury. Under normal physiological conditions, cellular adenosine flux is inwards due to its rapid intracellular metabolism to adenine nucleotides^8^. Inhibition of ENT1 (*SLC29A1*; equilibrative nucleoside transporter 1), the most studied of this family of transporters, causes vasodilation due to enhancement of the activity of adenosine at its receptors in the vasculature, and this has been shown to be cardioprotective^9–12^. However, ENT1 inhibitors have not proven effective clinically as they lead to vasodilation throughout the tissue, not just in the ischaemic regions; this can actually worsen the injury due to the ‘stealing’ of blood flow from the ischaemic areas^13,14^. Another consideration is that during ischaemia adenosine is generated intracellularly from the breakdown of adenine nucleotides and is then released from cells via equilibrative nucleoside transporters upon reperfusion of the tissue^15,16^. This loss of intracellular adenosine contributes to cellular dysfunction by limiting restoration of intracellular adenine nucleotide pools. Prevention of this loss of adenosine via transporter blockade would also be of benefit in ischaemia-reperfusion injury, but again, this would be of particular benefit if it was targeted to the damaged tissue. As hENT4 is known to be expressed in cardiomyocytes and vascular endothelial cells^1,3^, and its activity is increased under acidic conditions, inhibition of hENT4 may selectively enhance the ability of adenosine to protect the acidic vasculature in ischaemia as well as prevent the loss of adenosine from the cells during early reperfusion.

Previous work characterising hENT4 function as a nucleoside transporter has used [^3^H]adenosine as the substrate^3,17,18^. While adenosine is the physiological substrate, it is rapidly metabolised giving it an exceptionally short half-life^19^. This makes it difficult to differentiate between changes in intracellular metabolism and membrane transport kinetics as contributors to changes in the cellular accumulation of adenosine. This rapid intracellular conversion of adenosine to membrane impermeable adenine nucleotides also makes adenosine unsuitable for assessing the efflux kinetics of hENT4. Furthermore, these prior studies were done using hENT4-transfected Xenopus oocytes^3^ or MDCK cells^18^. Xenopus oocytes are not representative of a mammalian cell environment, and canine MDCK cells express ENT1 making it necessary to conduct the studies on hENT4 function in MDCK cells in the presence of the ENT1 blocker nitrobenzylthioinosine (NBMPR). NBMPR also blocks hENT4, albeit at concentrations considerably higher than those required for inhibition of ENT1^18^. To address these confounding issues, we assessed whether [^3^H]2-chloroadenosine, a poorly metabolized adenosine analogue^20^, is a substrate of hENT4 when expressed in the PK15-NTD cell line (a nucleoside transporter deficient variant of the swine kidney tubular epithelial cell line PK15)^21^. [^3^H]2-chloroadenosine has been used successfully for determining the kinetics of other nucleoside transporters such as ENT1 and ENT2^22–24^. In the present study, we show that 2-chloroadenosine is indeed a substrate for hENT4 that can be used to assess both the influx and efflux kinetics of this system. In addition, both the influx and efflux of 2-chloroadenosine by hENT4 are enhanced in an acidic environment.

## Materials and Methods

### Plasmid construction and transfection

The DNA sequence corresponding to the coding region of *SLC29A4* (NM_001040661) with an N-terminal myc-epitope tag was prepared by Integrated DNA Technologies (Edmonton, Canada) in the pUCIDT-AMP vector. This construct was transferred to pcDNA3.1 using the restriction enzymes XbaI (5′) and HindIII (3′). The pcDNA3.1 construct was propagated in E. Coli DH5α, extracted using the QIAprep Spin Miniprep Kit (Qiagen, Canada) and sequenced in the Alberta Transplant Applied Genomics Centre, Canada, to confirm identity, using the primers 5′-CCC AAG CTT GGC TCC GTG GGG AGC C-3′ (forward) and 5′-GCT CTA GAT CAG AGG CCT GCG AGG ATG-3′ (reverse). Approximately 1 × 10^6^ PK15-NTD^21^ cells grown in minimum essential media (MEM) containing no antibiotics were seeded into 16 well plates. The next day, 1.6 μg plasmid DNA and 4.8 µl LipofectAMINE 2000 (optimized ratio of 1:3) were each diluted into 100 µl Opti-MEM I Reduced Serum Medium and incubated at room temperature for 5 min. The mixtures were combined and incubated for an additional 20 min, then added to the PK15-NTD cells. After 24–48 h incubation, the media was replaced with MEM supplemented with sodium pyruvate (1 mM), non-essential amino acids (0.1 mM), penicillin (100 U/ml), streptomycin (100 μg/ml), 10% fetal bovine serum (FBS), 50 μg/ml nystatin, and 0.5 mg/ml G418. Selection pressure was maintained for 3 weeks, after which surviving cells were moved to 6-well plates with MEM supplemented as above but containing 0.3 mg/ml G418. Individual colonies (derived from a single cell) were allowed to proliferate to ~300 cells before being isolated using cloning cylinders and transferred to new 6-well plates. Expression of *SLC29A4* was confirmed by PCR analysis and myc-hENT4 protein expression by immunoblotting.

### Immunoblot

Samples were prepared in RIPA buffer (150 mM NaCl, 50 mM Tris, 1% NP-40, 0.5% sodium deoxycholate, and 1% SDS) containing a cocktail of protease inhibitors (MilliporeSigma, Oakville, Canada). Samples were adjusted to 2% (v/v) β-mercaptoethanol, and then resolved by SDS-PAGE on 12.5% (w/v) acrylamide gels. Proteins were electro-transferred onto Immobilon-P PVDF membranes (Millipore Corporation, MA, USA) for 1.5 h at a constant current of 280 mA. After transfer, membranes were rinsed in Tris-buffered saline (TBS; 0.15 M NaCl, 50 mM Tris, pH 7.5) and incubated with TBS containing 0.2% (v/v) Tween-20 and 5% (w/v) skim milk powder for 1 h at room temperature, with gentle rocking, to block nonspecific binding. Membranes were then incubated for 16 h at 4 °C, with gentle rocking, in the presence of either mouse anti-Myc (Clone 4A6, 05-724, Lot #2585792; EMD Millipore, Canada) or mouse anti-GAPDH (v-18, sc-20357, Lot #B2113; Santa Cruz Biotechnology, Canada) at 1:1000 and 1:500 dilutions, respectively, in TBS containing 0.2% (v/v) Tween-20 and 1% (w/v) skim milk. After successive washes with TBS (+0.2% Tween-20/1% skim milk), the membranes were incubated with a 1:3000 dilution of the appropriate HRP-conjugated secondary antibody (Donkey anti-goat IgG-HRP, sc-2020, Lot #A1013, or m-IgGk BP-HRP, sc-516102, Lot #F1016, both from Santa Cruz Biotechnology) in TBS (+0.2% Tween-20/1% skim milk), for 1 h at room temperature and further washed with TBS containing 0.2% (v/v) Tween-20. Proteins were detected using ECL western blot substrate (EMD Millipore, Canada) and visualized using a ChemiDoc™ XRS + System (Bio-Rad Laboratories, Canada).

### Polymerase Chain Reaction (PCR)

RNA was extracted from Trizol stabilized cells using chloroform phase separation. Briefly, 200 μl chloroform was added per ml of Trizol, the mixture vortexed, then centrifuged at 12,000 × g for 15 min at 4 °C. The upper phase was then transferred to a clean tube and mixed with 700 µl of isopropanol per ml of sample and centrifuged at 10,000 × g for 10 min at 4 °C. The supernatant was discarded and the pellet washed with 1 ml of 75% ethanol (with centrifugation at 7,500 × g for 5 min at 4 °C). This supernatant was discarded and the pellet air-dried for 5 min and suspended in 100 µl RNase/DNase-free water. Total RNA content and purity was determined using a Nanodrop 2000 spectrophotometer (Life Technologies Inc., Burlington, ON, Canada). First-strand complementary DNA (cDNA) was synthesised from 1 μg of total RNA using M-MLV reverse transcriptase (Invitrogen). PCR was performed using *Taq* DNA Polymerase (ThermoFisher) in a T100™ Thermal Cycler (Bio-Rad Laboratories, Canada). Samples were heated to 95 °C for 3 min, 40 cycles of 30 s at 95 °C, 30 s at 58 °C, and 60 s at 72 °C, followed by 72 °C for 15 min. *SLC29A4* transcript was amplified using the following primers: 5′- CTG GAG CTG CTG TGT TTC CT -3′ (forward), 5′- CAG TAA CAG GGC TCT GAA GG -3′ (reverse).

### [^3^H]Substrate uptake

*SLC29A4*-transfected PK15-NTD cells (PK15-hENT4), and the un-transfected PK15-NTD cells, were seeded in 6-well plates and allowed to grow for 2 days. Upon reaching 80–90% confluency, culture media was removed and plates washed with PBS (137 mM NaCl, 2.7 mM KCl, 10 mM Na_2_HPO_4_, 1.8 mM KH_2_PO_4_, pH 7.4) twice and then incubated in room temperature (~22 °C) transport buffer (120 mM NaCl, 20 mM Tris, 3 mM K_2_HPO_4_, 10 mM glucose, 1 mM CaCl_2_, 1 mM MgCl_2_, pH 7.5) for 15 min. Time courses of substrate uptake were constructed by exposing the cells to the substrate ([^3^H]adenosine or [^3^H]2-chloroadenosine) in room temperature transport buffer, pH 6.0 or pH 7.5, for times ranging from 1 to 15 min. 30 µM was chosen as the substrate concentration to use for the initial time course studies, as this is within the range of local adenosine concentrations achieved pathologically in ischaemic tissues. Uptake was terminated by rapidly aspirating the reaction mixture from the plates and washing the cells thrice with ice-cold PBS. Cells were digested in 1 N sodium hydroxide overnight, and radioactive content of each sample was measured using standard liquid scintillation techniques. Samples of the cell digest from each well were also taken for assessment of protein content for intra- and inter-assay normalisation. hENT4-mediated uptake was defined as the difference between total uptake by the PK15-hENT4 cells (at pH 6.0 or 7.5) and that measured in parallel using the PK15-NTD cells (at the corresponding pH). Uptake by the un-transfected PK15-NTD cells, which are devoid of ENT1 and ENT2 transport activity^21,25^, showed no acidic pH-dependent accumulation of [^3^H]nucleosides and was thus considered to represent “background” non-transporter mediated accumulation. Based on initial time course profiles, it was determined that a time point of 5–6 min provided a reasonable estimate of the initial rate of [^3^H]substrate influx. Therefore, this time frame was used to obtain initial rates of uptake over a range of concentrations of substrate to define transporter kinetics.

### [^3^H]2-Chloroadenosine efflux

PK15-hENT4 cells in 6-well plates were loaded with 30 μM [^3^H]2-chloroadenosine in transport buffer, at pH 6.0, for 12 min, in the presence of 50 nM ABT-702 to minimize metabolism by adenosine kinase (concentrations and optimum loading times determined from the substrate uptake experiments). After loading, cells were washed thrice with ice-cold PBS to remove remaining extracellular [^3^H]substrate. To estimate the amount of extracellular [^3^H] remaining on the plates after this wash procedure, the washed cells were exposed to 800 µl of ice-cold buffer for ~10 s and the [^3^H] content of a 500 µl aliquot of this wash media assessed; this ‘background’ was subtracted from all efflux data. For the efflux time courses, efflux was initiated by adding room temperature (~22 °C) substrate-free buffer (800 µl), pH 6.0, 7.5 or 8.2 (±test inhibitors) to the plates. After a defined incubation time (1–18 min), 500 µl of the extracellular media was removed and assessed for [^3^H] content. Cell protein content of each well was also measured and used for assay normalisation as described for the [^3^H]substrate uptake assays.

### Intracellular pH (pH_i_) measurement

pH_i_ was measured using a PTI Deltascan spectrofluorometer^26^. Cells were grown on glass coverslips (Thomas® RedLabel® Micro Cover Glasses) to ~90% confluency. Cells were loaded with BCECF-AM (20,70-bis(2-carboxyethyl)-5(6) carboxyfluorescein-acetoxymethyl ester) (Molecular Probes Inc., Eugene, OR, USA) at a final concentration of 2.5 µg/ml (1 µl of 1 mg/ml stock in DMSO in 400 µl serum-free culture media at 37 °C for approximately 20 min). The AM group is de-esterified during this incubation, trapping BECEF within the cell. A dual excitation (440 and 502.5 nm), single emission (528.7 nm) ratio ensures that measurement is independent of cell number or amount of BECEF inside the cells. After loading, the coverslip was transferred to a cuvette holder in transport buffer, pH 7.5, with constant stirring by a magnetic stir bar at room temperature for 15 min. Following which, incubation times and conditions used for [^3^H]2-chloroadenosine uptake assays were applied to determine how pH_i_ changes during uptake at the different pH conditions used (6.0, 7.5, 8.2). For each experiment, a three-point pH calibration curve was made with a high KCl transport buffer (120 mM NaCl was replaced by 120 mM KCl) and 10 μM nigericin.

### Statistical analysis

Experiments were repeated at least five times in duplicate (actual N values reported in the Figure legends). Data are expressed as mean ± S.E.M. Statistical significance was determined by Student’s *t* test or one-way analysis of variance (ANOVA) plus an appropriate multiple comparisons post-test, as indicated in the Figure legends, with P < 0.05 denoting significance. GraphPad Prism v8.01 was used to define the best fit (F-test, P < 0.05) nonlinear relationship represented by the data, and for all statistical analyses.

## Results

### SLC29A4/ENT4 expression

**Figure 1:**
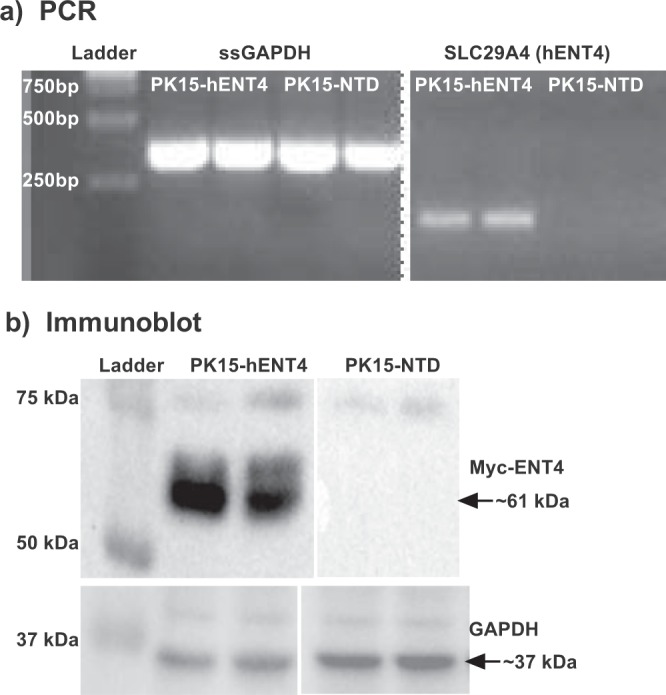
SLC29A4/hENT4 expression in PK15-NTD cells and *SLC29A4* transfected PK15-NTD cells. (**a**) PCR: cDNA was prepared from Total RNA isolated from PK15-NTD cells and cells stably transfected with *SLC29A4* (PK15-hENT4). The transcripts for transfected human *SLC29A4* and endogenous pig GAPDH were amplified as described in the text. (**b**) Immunoblots: Membranes were prepared from PK15-NTD and PK15-hENT4 cells. Samples were resolved on SDS-PAGE gels, transferred to polyvinyl membranes and probed with anti-myc and anti-GAPDH antibodies to detect myc-tagged hENT4 and GAPDH (loading control), respectively. Complete gels are shown in Supplemental Fig. 1.

PK15-NTD cells do not have any detectable transcript for *SLC29A4*, whereas *SLC29A4* expression was evident using RNA isolated from PK15-NTD cells stably transfected with *SLC29A4* (Fig. 1a). Myc-ENT4 protein was detected in the *SLC29A4* transfected cells by immunoblotting with an anti-myc antibody, and had a molecular mass of approximately 61 kDa (Fig. 1b). The predicted molecular mass of hENT4 is ~55 kDa, suggesting that the recombinant hENT4 protein expressed in these cells may be glycosylated (hENT4 is predicted to be glycosylated at N523 near the C-terminus).

### [^3^H]Adenosine uptake

**Figure 2:**
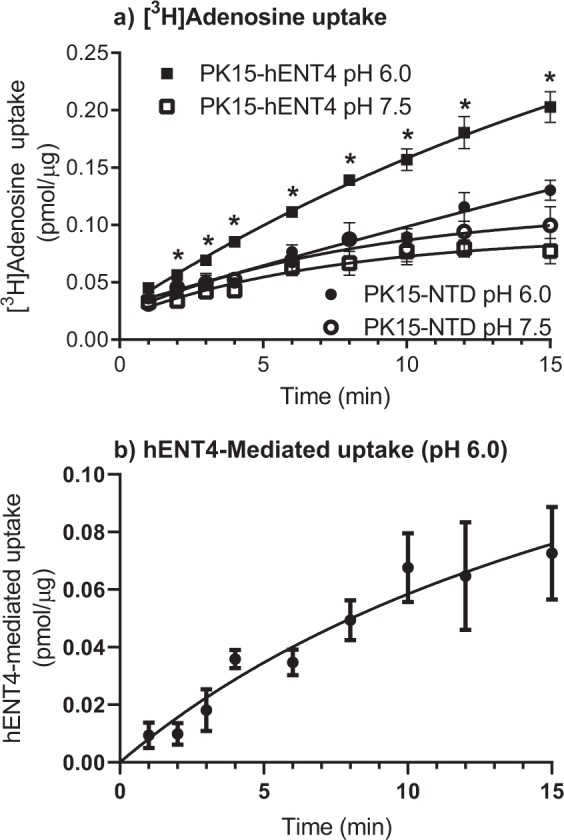
[^3^H]Adenosine uptake by hENT4: (**a**) PK15-NTD and PK15-hENT4 cells were incubated with 30 μM [^3^H]adenosine at ~22 °C for the specified times in the presence of the adenosine kinase inhibitor ABT-702 (50 nM) and the adenosine deaminase inhibitor EHNA (100 nM). *Significant difference in uptake by PK15-hENT4 cells at pH 6.0 versus pH 7.5 (Two way ANOVA with Tukey’s multiple comparison test, P < 0.05, n = 5). Note that there was no significant difference in uptake by the PK15-NTD cells at pH 6.0 versus pH 7.5. (**b)** hENT4-mediated uptake of [^3^H]adenosine was calculated from the data shown in Panel a as the difference in uptake between PK15-hENT4 and PK15-NTD cells at pH 6.0. For both Panels, data fit best to a one-phase association curve.

**Figure 3:**
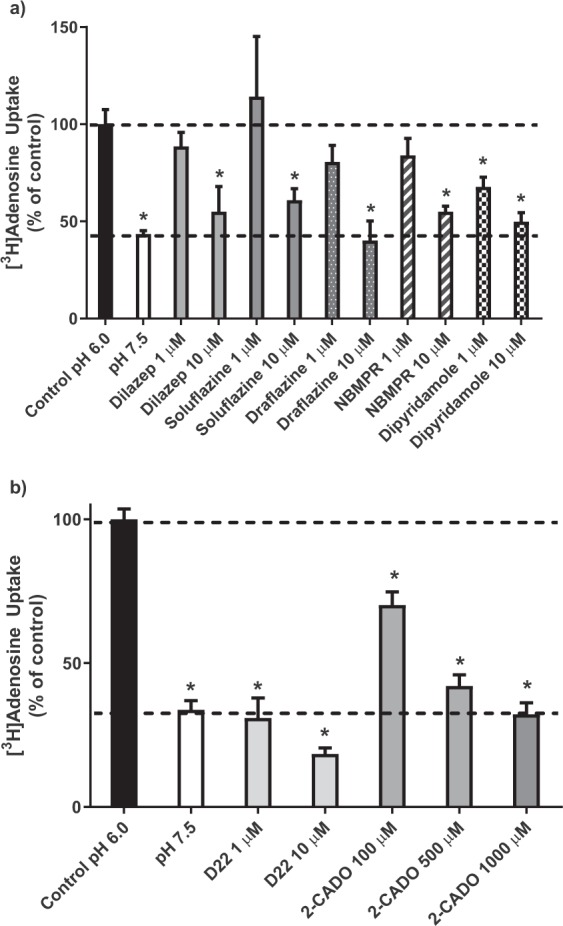
Inhibition of hENT4-mediated [^3^H]adenosine uptake. Uptake of 1 µM [^3^H]adenosine by PK15-hENT4 cells (6 min incubation) was assessed at pH 6.0 in the presence and absence of the indicated concentrations of established inhibitors of ENT1 and ENT2 (Panel a), or the hENT4 inhibitor D22 and the adenosine analogue 2-chloroadenosine (2-CADO) (Panel b). Uptake in the absence of inhibitors at pH 7.5 is also shown. Data are expressed as a percentage of the uptake measured at pH 6.0 in the absence of inhibitor (Control). Each bar is the mean ± S.E.M. from 5 experiments conducted in duplicate. *Significantly different from Control (One way ANOVA with Dunnett’s multiple comparisons test, P < 0.05).

Initial studies were conducted to confirm that hENT4 mediated the uptake of [^3^H]adenosine in an acidic pH-sensitive manner, as described previously^3,18^. These assays were done in the presence of 50 nM ABT-702 to inhibit adenosine kinase and 100 nM erythro-9-(2-hydroxy-3-nonyl)adenine (EHNA) to inhibit adenosine deaminase. In all cases, time course data fit best to a one-phase (versus two-phase) association profile (Equation: Y = Y0 + (Plateau-Y0)*(1-exp(-K*x), with Y0 fixed to zero for fitting mediated uptake data, but left variable when fitting total uptake data with no background subtracted). The uptake of 30 µM adenosine by the PK15-hENT4 cells was significantly greater at pH 6.0 versus pH 7.5, whereas no significant difference with pH was seen for adenosine uptake by the un-transfected PK15-NTD cells (Fig. 2a). In addition, there was no significant difference in adenosine uptake between PK15-hENT4 cells and PK15-NTD cells at pH 7.5. The near linear accumulation of [^3^H] by the cells at pH 6.0 even at 15 min of incubation suggests that there continues to be some trapping of [^3^H]adenosine metabolites (adenine nucleotides) by the cells over this time frame even in the presence of the adenosine kinase inhibitor ABT-702. However, given that caveat, when the adenosine uptake by the PK15-NTD cells at pH 6.0 was subtracted from uptake by the PK15-hENT4 cells at pH 6.0, an initial rate of hENT4-mediated uptake of 9 pmol/mg/min could be estimated based on the maximum accumulation (187 ± 63 pmol/mg) and t_1/2_ (22 ± 11 min) derived from the fitted curve (Fig. 2b). Inhibitors of other SLC29A family members (ENT1/ENT2) were then tested for their ability to inhibit the uptake (6 min) of 1 μM [^3^H]adenosine at pH 6.0 (Fig. 3a). NBMPR, dilazep, draflazine, and soluflazine all produced a significant inhibition of [^3^H]adenosine uptake by hENT4 when tested at 10 µM, but had no effect at 1 µM. These inhibitors would be expected to inhibit ENT1- and/or ENT2-mediated nucleoside uptake to some extent at the concentrations used^21,25,27,28^. Dipyridamole was the only compound tested in this set with significant inhibitory activity against hENT4 at 1 µM (Fig. 3a). In comparison, the hENT4 inhibitor decynium 22 (D22; 1 μM)^29^ completely blocked the acidic pH-dependent component of [^3^H]adenosine uptake (Fig. 3b). Higher concentrations of D22 (10 µM) appeared to affect uptake at pH 7.5, but it was not statistically significant in this data set. Finally, if 2-chloroadenosine is a substrate for hENT4, one would expect it to compete with [^3^H]adenosine for the substrate binding site on the hENT4 transporter. As shown in Fig. 3c, 2-chloroadenosine had a concentration-dependent inhibitory effect on [^3^H]adenosine uptake by the PK15-hENT4 cells, with complete inhibition of the acidic pH-dependent component achieved with 1 mM 2-chloroadenosine.

### [^3^H]2-chloroadenosine uptake

**Figure 4:**
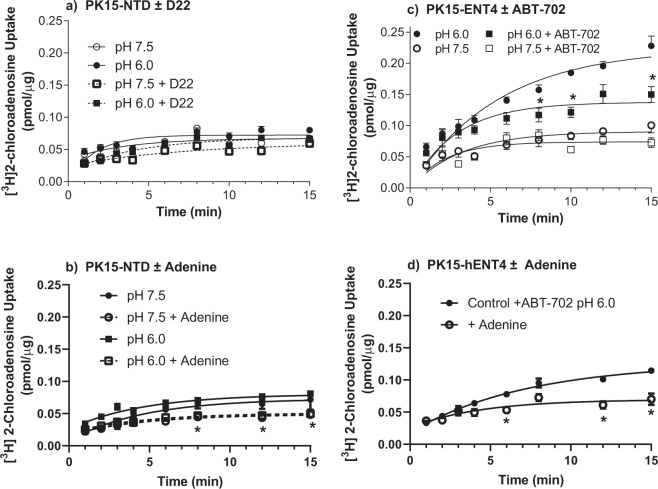
Time course of [^3^H]2-chloroadenosine uptake by PK15-NTD and PK15-hENT4 cells. Cells were incubated at ~22 °C for the specified times with 30 µM [^3^H]2-chloroadenosine in either pH 7.5 or pH 6.0 buffer in the presence and absence of the indicated inhibitors (Panel a: PK15-NTD ± 10 µM D22, an established hENT4 inhibitor; Panel b: PK15-NTD ± 10 mM adenine; Panel c: PK15-hENT4 ± 50 nM ABT-702, an adenosine kinase inhibitor; Panel d: PK15-hENT4 cells ± 10 mM adenine in the presence of ABT-702. Each point represents the mean ± S.E.M. from 5 experiments conducted in duplicate, and one-phase association curves were fitted to the data as shown. *Denotes a significant difference in uptake in the presence of the modifier (D22, ABT-702, or adenine) versus its absence at the respective pH (Two way ANOVA with Tukey’s multiple comparison test, P < 0.05).

**Figure 5:**
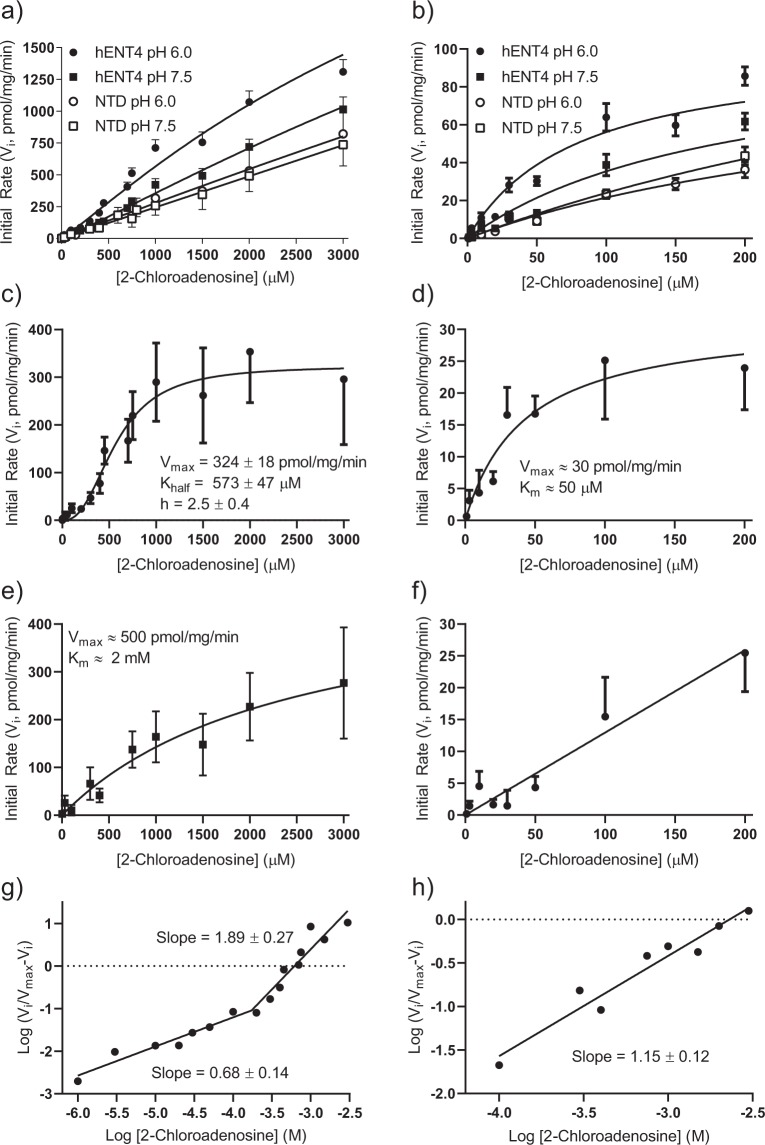
Concentration-dependence of [^3^H]2-chloroadenosine uptake. PK15-NTD and PK15-hENT4 cells were exposed to the indicated concentrations of [^3^H]2-chloroadenosine for 5 min at pH 7.5 or pH 6.0, in the presence of 50 nM ABT-702. Each point represents the mean ± S.E.M of at least 6 independent experiments conducted in duplicate. Panel a shows the data for the full concentration range used. Panel b is an expanded view of the data for the concentration range of 1–200 µM from Panel a. Panel c: To assess the kinetics of the acidic pH-dependent transport component, uptake (from Panel a) by PK15-hENT4 cells at pH 7.5 was subtracted from that obtained at pH 6.0 The resulting data set was best described by an allosteric sigmoidal non-linear model with the kinetic parameters shown. Panel d is an expanded view of the data for the concentration range of 1–200 µM in Panel c. Data in this range were best described by a non-cooperative Michaelis-Menten relationship. Due to the large errors that resulted from the data subtraction, only approximate kinetic constants could be obtained in this case. Panel e: To assess the kinetics of the ENT4-mediated transport at pH 7.5, uptake (from Panel a) by PK15-NTD cells at pH 7.5 was subtracted from that using the PK15-hENT4 cells at pH 7.5. This transport component fit best to a regular Michaelis-Menten relationship. Panel f is an expanded view of the data for the concentration range of 1–200 µM in Panel e. These data were best described by a linear relationship. Panels g and h are the Hill Plot derivations of the data shown in Panels c and e, respectively. The slopes of the fitted lines, which represent the Hill coefficients, are indicated.

As for [^3^H]adenosine, the [^3^H]2-chloroadenosine uptake time course fit best to a one-phase (versus two-phase) association profile. There was no significant difference in [^3^H]2-chloroadenosine uptake by the PK15-NTD cells between pH 7.5 and 6.0 (Fig. 4a), in either the presence or absence of the hENT4 blocker D22. These findings, along with the lack of acidic pH-sensitive [^3^H]adenosine uptake (Fig. 2a) by PK15-NTD cells, and no detectable mRNA transcript in the PK15-NTD cells (Fig. 1a), indicates that there is no endogenous ENT4 activity in this cell line. Therefore, since the PK15-NTD cells are also devoid of ENT1, ENT2, and concentrative Na-dependent nucleoside transporters^21,25^, enhanced 2-chloroadensine accumulation upon transfection of the PK15-NTD cells with *SLC29A4* may be considered hENT4-mediated. A potential complicating factor, however, is the reported presence of an equilibrative nucleobase transporter (ENBT1) in the PK15-NTD cells for which 2-chloroadenosine may have some affinity^30,31^. To address this, we assessed the ability of adenine, a substrate for ENBT1 but not ENT4^18^, to inhibit 2-chloroadenosine uptake by PK15-NTD cells at both pH 7.5 and 6.0. Adenine, at concentrations sufficient to completely block ENBT1 activity (10 mM) had a slight suppressant effect on the uptake of 30 µM 2-chloroadenosine at both pH 6.0 and pH 7.5, with the difference at pH 6.0 achieving statistical significance (Fig. 4b). The acidic pH sensitivity of [^3^H]2-chloroadenosine uptake was then assessed using the *SLC29A4*-transfected PK15-NTD cells (Fig. 4c). There was no significant difference in 2-chloroadenosine (30 µM) uptake between PK15-hENT4 and PK15-NTD cells at pH 7.5. In contrast, at pH 6.0, PK15-hENT4 cells accumulated 30 µM [^3^H]2-chloroadenosine at a significantly greater rate than seen at pH 7.5 in either the PK15-hENT4 or PK15-NTD cells. However, as observed for [^3^H]adenosine, the cellular accumulation of [^3^H] using [^3^H]2-chloroadenosine as the substrate did not saturate even at the longest incubation time of 15 min. To determine whether this represented trapping of radiolabel due to the formation of impermeable [^3^H]2-chloroadenosine metabolites, the adenosine kinase inhibitor ABT-702 was added. ABT-702 (50 nM) reduced the total accumulation of [^3^H] from the [^3^H]2-chloroadenosine leading to saturation of the cellular uptake profile by 15 min (Fig. 4b). This suggests, that over this extended time of incubation, 2-chloroadenosine was being metabolised by adenosine kinase resulting in the trapping of [^3^H] as phosphorylated [^3^H]2-chloroadenosine derivatives. ABT-702 was therefore used in all subsequent experiments to minimise metabolism of 2-chloroadenosine by adenosine kinase. Figure 4d shows that 10 mM adenine also decreased 2-chloroadenosine uptake in the PK15-hENT4 cells to a degree similar to that seen for the PK15-NTD cells (Fig. 4b), likely due to the activity of ENBT1 in these cells. When [^3^H]2-chloroadenosine uptake at pH 6.0 in the PK15-NTD cells (which includes the ENBT1-mediated uptake component) was subtracted from uptake at pH 6.0 in the PK15-hENT4 cells, the initial rate of hENT4-mediated [^3^H]2-chloroadenosine (30 µM) uptake was estimated as 8 pmol/mg/min with a maximum accumulation of 124 ± 65 pmol/mg and a t_1/2_ of 16 ± 11 min. This is similar to the initial rate of hENT4-mediated uptake defined above for 30 µM [^3^H]adenosine. The uptake of a range of concentrations of [^3^H]2-chloroadenosine up to 3 mM (solubility limit), in the presence of ABT-702, was then assessed using a 5 min incubation time (approximates initial rate of influx) in PK15-NTD cells and PK15-hENT4 cells at pH 7.5 and 6.0 (Fig. 5a,b). Uptake by the PK15-NTD cells was linear with concentration, indicative of a non-mediated process, and there was no significant difference in uptake by PK15-NTD cells at pH 6.0 versus pH 7.5. However, in addition to the enhanced uptake at pH 6.0 in the PK15-hENT4 cells, there was also significantly more uptake in the PK15-hENT4 cells at pH 7.5 when compared with uptake by the PK15-NTD cells at pH 7.5, particularly at the higher concentrations of substrate (>750 µM). These data sets were then analyzed to obtain the acidic pH-dependent transport component (Fig. 5c,d; difference in uptake by PK15-hENT4 cells at pH 6.0 versus pH 7.5) and the component that is not dependent on an acidic pH (Fig. 5e,f; PK15-hENT4 minus PK15-NTD, pH 7.5). Analyses of these data sets revealed that the acidic-pH-dependent transport component (Fig. 5c) was best described (F-test, P < 0.05) by a allosteric sigmoidal relationship (Y = V_max_*X^h/(K_half_^h + X^h) where V_max_ is the maximum transport velocity for the substrate, K_half_ is the concentration of substrate that produces half-maximal transport velocity (EC_50_), and h is the Hill coefficient. The acidic pH-dependent component described in Fig. 5b had a V_max_ of 324 ± 18 nmol/µg/min, a K_half_ of 573 ± 47 µM, and a Hill coefficient of 2.5 ± 0.4 which is indicative of positive cooperativity. The Hill Plot derivation of these data show a significantly biphasic relationship (Fig. 5g), with a Hill coefficient of 0.68 ± 0.14 at substrate concentrations less than 200 µM and a Hill coefficient of 1.89 ± 0.27 at higher concentrations. The latter Hill coefficient is significantly greater than one, which is also indicative of positive cooperativity. When only the low concentrations of 2-chloroadenosine (1–200 µM) were considered, representing the portion of the acidic pH-dependent substrate/rate profile more reflective of endogenous adenosine concentrations, the acidic-pH dependent transport data fit best to a simple non-cooperative Michaelis-Menten relationship (Fig. 5d) with a V_max_ of ~30 pmol/mg/min and a K_m_ ~50 µM. Note that due to the large variability that results from the data subtraction process used to obtain this sub-component, only approximations of the kinetic constants can be derived. In contrast, the hENT4-mediated transport component that remained at neutral pH (Fig. 5e) was best described by a simple non-cooperative Michaelis-Menten relationship (Y = V_max_*X/(K_m_ + X)), giving a V_max_ of ~500 pmol/mg/min, a K_m_ of ~2 mM (with no evidence of a high affinity component (Fig. 5f), and a Hill coefficient not significantly different from 1 (1.15 ± 0.12, Fig. 5h).

### Inhibition of hENT4-mediated nucleoside uptake

**Figure 6:**
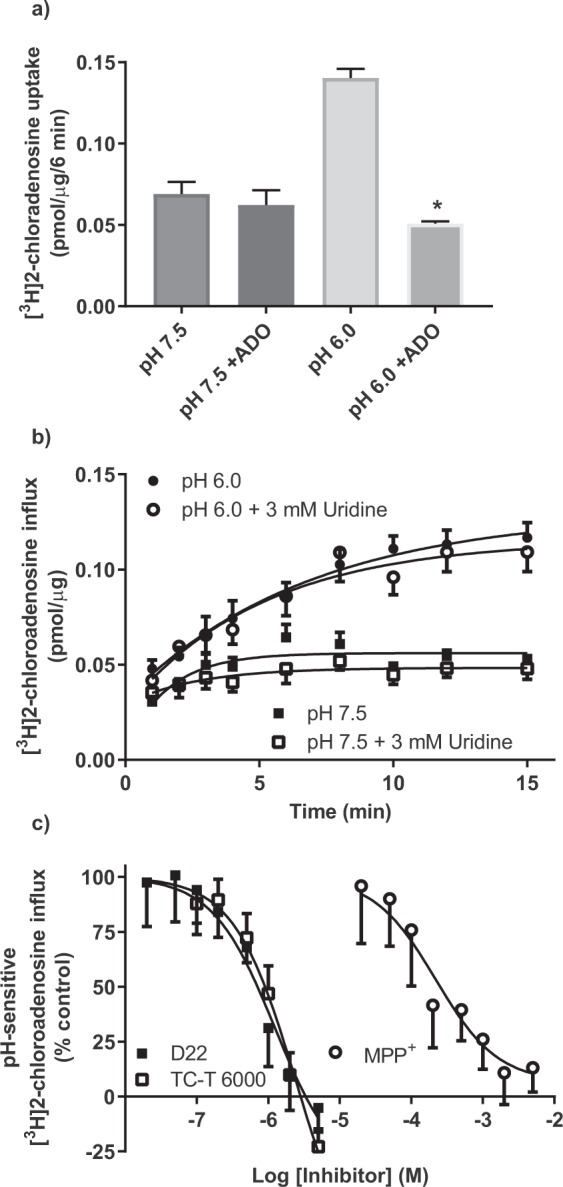
Inhibition of [^3^H]2-chloroadenosine influx in PK15-hENT4 cells. (**a**) The uptake of 30 µM [^3^H]2-chloroadenosine (6 min incubation) was assessed at pH 7.5 and pH 6.0 in the presence and absence of 10 mM adenosine (ADO). Each bar represents the mean ± S.E.M. from 5 experiments conducted in duplicate. *Significant difference ± ADO at pH 6.0 (One way ANOVA with Tukey’s multiple comparisons post-test, P < 0.05). (**b)** The uptake of 30 µM [^3^H]2-chloroadenosine was assessed at the indicated times at pH 7.5 and pH 6.0 in the presence and absence of 3 mM uridine (ADO). Each point represents the mean ± S.E.M. from 5 experiments conducted in duplicate. Note that there is no significant effect of uridine on 2-chloroadenosine uptake at either pH 7.5 or pH 6.0. (**c)** The uptake of 30 µM [^3^H]2-chloroadenosine (6 min incubation) was assessed at pH 6.0 in the presence and absence of the indicated concentrations of D22, TC-T6000 or MPP^+^. Data are shown as % of control where 100% was the uptake at pH 6.0 in the absence of inhibitor and 0% was the uptake measured at pH 7.5 in the absence of inhibitor. Each point represents the mean ± S.E.M from 5 (D22, TC-T6000) or 6 (MPP^+^) experiments conducted in duplicate. Data shown were fitted by sigmoid curves with the maximum constrained to 100% but with no constraint on the minimum.

Adenosine significantly inhibited the hENT4-mediated uptake of 30 µM [^3^H]2-chloroadenosine at pH 6.0 but not at pH 7.5 (Fig. 6a). The lack of effect of adenosine on at pH 7.5 likely reflects the relatively low concentration of [^3^H]2-chloroadenosine used for this particular assay. hENT4-mediated uptake at neutral pH is only apparent at higher substrate concentrations (see Fig. 5). Uridine, on the other hand, which can inhibit all of the other known purine nucleoside transporters (ENT1, ENT2, CNT2, CNT3) in the low- to mid-µM range^32,33^, did not inhibit hENT4 at concentrations up to 3 mM (Fig. 6b). This suggests that 3 mM uridine may be used to block mediated uptake of [^3^H]2-chloroadenosine by other nucleoside transporters without inhibiting hENT4 in cell populations that express multiple nucleoside transporter subtypes. This lack of effect of uridine on [^3^H]2-chloroadenosine uptake by PK15-hENT4 cells, along with the insensitivity of [^3^H]adenosine uptake in the PK15-hENT4 cells to known inhibitors of ENT1 and ENT2 (Fig. 3a), also confirms that the base PK15-NTD cells are indeed deficient in all nucleoside transporter activity. The known hENT4 substrate MPP^+^ inhibited 30 µM 2-chloroadenosine uptake with an IC_50_ of 220 µM (log IC_50_ = −3.66 ± 0.11). An unconstrained sigmoid curve fit to the MPP^+^ data indicated that MPP^+^ appeared to inhibit only the acidic pH-dependent component of the uptake (extrapolated lower limit of 6.5 ± 6.2% of control). This is in contrast to what was observed when using D22 or the more hENT4-selective analogue of dipyridamole, TC-T6000^17^. These agents appeared to inhibit both the acidic pH-dependent uptake and the hENT4-mediated uptake of 2-chloroadenosine at neutral pH (based on the observation that the inhibition profiles did not plateau at 0% in Fig. 6c). A trend in this regard was also seen for 10 µM D22 (Fig. 3b). While it is not possible to realistically determine the base of the inhibition profile from the data shown, it can be estimated from the results shown in Fig. 5a that about 55% of the uptake of 30 µM 2-chloroadenosine by the PK15-hENT4 cells is dependent upon an acidic pH. Therefore, inhibition of all hENT4-mediated uptake should theoretically plateau at about −82% on the graph shown in Fig. 6c. Thus, constraining a sigmoid curve fit to −82% allows a rough estimation of an IC_50_ value of 2.3 µM for both D22 (logIC_50_ = −5.64 ± 0.05) and TC-T6000 (logIC_50_ = −5.63 ± 0.04). These IC_50_ values for MPP^+^, D22, and TC-T6000 are comparable to those determined previously^17,18,34^, particularly when one considers the different expression models and substrate concentrations used.

### hENT4-mediated [^3^H]2-chloroadenosine efflux

**Figure 7:**
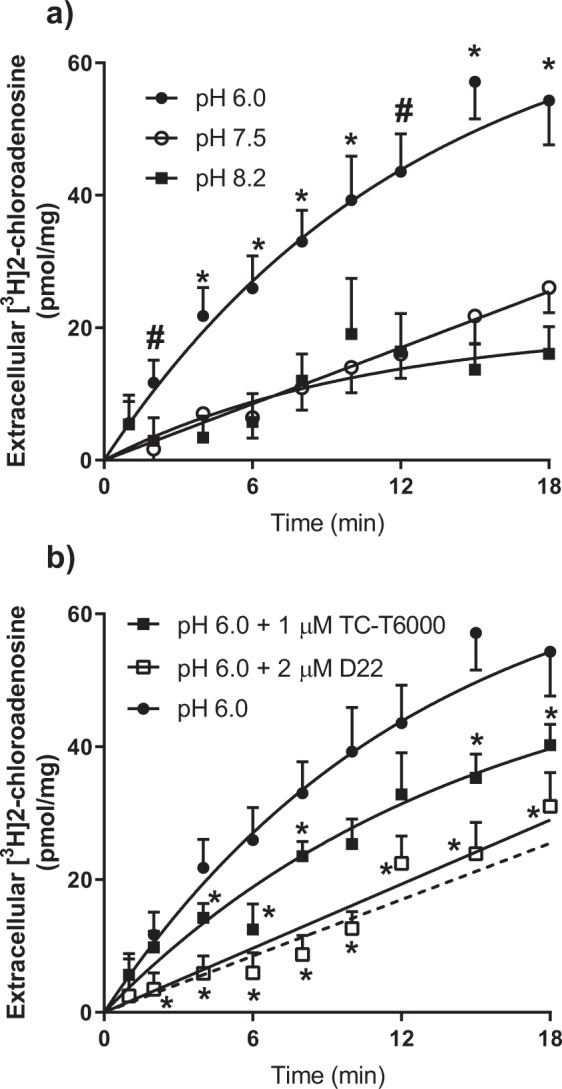
hENT4-mediated efflux of [^3^H]2-chloroadenosine. (**a**) PK15-hENT4 cells were incubated with 30 μM [^3^H]2-chloroadenosine and 50 nM ABT-702 for 12 min in pH 6.0 transport buffer. Cells were washed in ice-cold buffer to remove extracellular substrate, and then efflux was initiated by adding transport buffer at the indicated pH. Aliquots of extracellular solution were taken at the specified times and assessed for [^3^H]content. Data were fitted by a one-phase association curve. Each point represents the mean ± S.E.M. from 5 independent experiments. *Significant difference between efflux at pH 6.0 and 7.5 or 8.2. # Significant difference between pH 6.0 and 7.5 (Two-way ANOVA with Tukey’s multiple comparisons test, P < 0.05). (**b)** Inhibition of efflux by D22 and TC-T6000. [^3^H]2-chloroadenosine efflux was measured at pH 6.0, as described for Panel a, in the presence of D22 (2 μM) or TC-T6000 (1 μM). Each bar represents the mean ± S.E.M. from 5 experiments. The dashed line represents the uptake at pH 7.5 (from Panel a), shown for comparison. *Significant difference from efflux at pH 6.0 in the absence of inhibitor (Two way ANOVA with Tukey’s multiple comparisons test, P < 0.05).

PK15-hENT4 cells preloaded with [^3^H]2-chloroadensine (in the presence of the adenosine kinase inhibitor ABT-702) released [^3^H]2-chloroadenosine at an initial rate (curve fits shown in Fig. 7a extrapolated to 10 s) of 6.9 ± 1.5 pmol/mg/min when efflux was measured at a pH of 6.0. This rate of efflux was decreased significantly (One way ANOVA with Dunnett’s multiple comparisons test, P < 0.05) when the pH of the buffer was 7.5 (1.6 ± 0.2 pmol/mg/min) or 8.2 (2.5 ± 0.5 pmol/mg/min) (Fig. 7a). There was no significant difference in the rates of efflux at pH 7.5 and 8.2. hENT4-mediated efflux at pH 6.0 in the presence of 2 μM D22 was not significantly different from efflux at pH 7.5 (Fig. 7b), suggesting complete inhibition of the acidic pH-dependent efflux by D22. TC-T6000 (1 µM) also produced significant inhibition (~60%) of the acidic-pH dependent 2-chloroadenosine efflux (Fig. 7b).

### Measurement of intracellular pH

**Figure 8:**
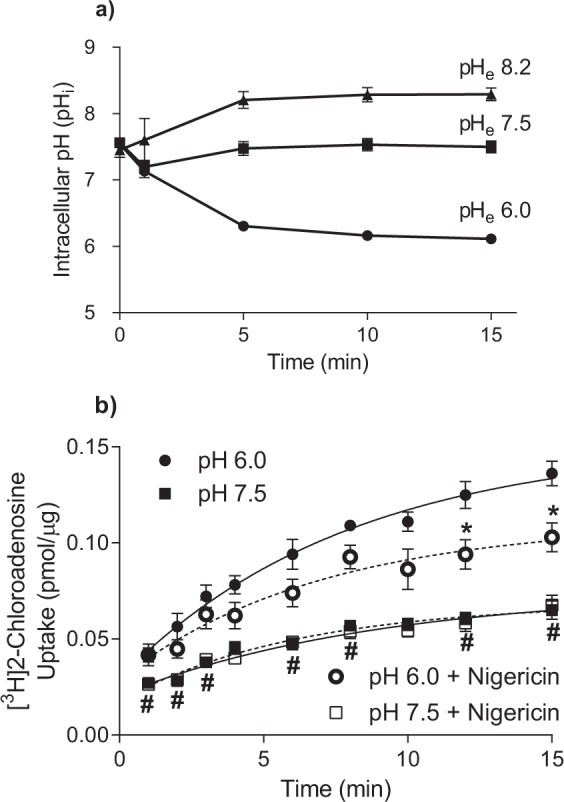
Proton gradient stability and effect of proton ionophore nigericin: (**a)** Cells grown on glass coverslips were loaded with BCECF-AM and placed in transport buffer at the indicated extracellular pH (pHe). Intracellular pH was assessed over the indicated time period by ratiometric fluorescence. Each point is the mean ± S.E.M. of 5 independent experiments. (**b)** [^3^H]2-chloroadenosine uptake was measured over time at pH 6.0 and pH 7.5 in the presence and absence of the proton ionophore nigericin (10 µM). Each point is the mean ± S.E.M. from 6 experiments conducted in duplicate. *Significantly different from pH 6.0 in the absence of nigericin. ^#^Significantly different from pH 7.5, with or without nigericin (Two way ANOVA with Tukey’s multiple comparison test, P < 0.05).

For the transport studies described above, cells were incubated in solutions of different pH for up to 30 min (15 min pre-incubation for uptake studies +15 min substrate uptake/efflux time course), with the intent being to establish a proton gradient across the cell membrane. However, measurement of intracellular pH during these incubation conditions established that the initial pH gradient across the cell membrane declined rapidly when cells were incubated in either pH 6.0 or pH 8.2 buffer, with the intracellular pH equilibrating with the extracellular pH within 5 min (Fig. 8a), which was the time point used to assess transporter kinetics at pH 6.0 versus 7.5 in this study (Fig. 5). The requirement of a proton gradient for the acidic pH-dependent uptake by hENT4 was also examined by assessing the rate of influx of hENT4-mediated [^3^H]2-chloroadenosine (30 µM) in the presence and absence of the proton ionophore nigericin (10 µM). It was confirmed via intracellular pH analyses that this concentration of nigericin completely collapsed the proton gradient in these cells. Nigericin had no effect on the uptake of [^3^H]2-chloroadenosine at a pH of 7.5 (Fig. 8b). With a pH of 6.0, nigericin treatment did result in a significant decrease in 2-chloroadenosine influx at the longer time points (affecting maximum accumulation but not influx rate), but it was still significantly higher than that observed at pH 7.5 (Fig. 8b) indicating that a complete collapse of the proton gradient did not eliminate the acidic pH effect on hENT4-mediated transport.

## Discussion

Our results show that ENT4 mediates both the influx and efflux of 2-chloroadenosine, confirming that hENT4 is a bidirectional transporter for nucleosides like other members of the ENT family^35–38^. While adenosine uptake by hENT4 has been reported previously^18^, this is the first study to show that hENT4 can also transport adenosine analogues. We also confirm previous reports that substrate uptake by hENT4 is enhanced by acidic pH^18^. A novel finding, though, is that the acidic pH-dependent component of 2-chloroadenosine uptake by hENT4 displays positive cooperativity (Hill coefficient of ~2) at substrate concentrations in excess of 200 µM. Furthermore, at lower substrate concentrations, in the more physiological range (<200 µM), hENT4, at acidic pH, is similar to other ENT subtypes with a K_m_ for adenosine of ~50 µM^24,39^. This supports the relevance of hENT4 in mediating the transmembrane flux of nucleosides in the acidic environments associated with conditions such as ischaemia-reperfusion injury^40–42^. Given that ENT1 and ENT2 function is suppressed in hypoxic tissue^43–45^, hENT4 may assume an even more dominant role in mediating adenosine flux in these conditions. In contrast, the component of hENT4-mediated transport that is not dependent on an acidic pH does not show cooperativity and follows simple Michaelis Menten kinetics with a much lower affinity for 2-chloroadenosine (K_m_ ~ 2 mM, Hill coefficient = 1.15). Therefore, acidification of the environment of hENT4 enhances the affinity of 2-chloroadenosine for the transporter. The appearance of this high affinity component at acidic pH also explains why most of the uptake observed in the initial time course experiments using 30 µM 2-chloroadenosine appeared predominantly acidic pH-dependent (Figs 3, 4 and 6). Whether this also occurs for the endogenous substrate adenosine has yet to be determined, but the attainment of such detailed transporter kinetics for adenosine would be problematic due to its rapid metabolism. The mechanism(s) underlying the change in substrate affinity and the positive cooperativity in this system at the higher substrate concentrations is unknown at present. Further analysis will require a model that has a higher level of hENT4 expression and lower background uptake (improving ‘signal:noise’ ratio) than the current PK15-ENT4 model provides. One can speculate that it may reflect allosteric changes in transporter protein structure due to multiple substrate binding sites with different affinities, or it may involve substrate driven changes in transporter oligomerization. Other nucleoside transporters such as ENT1 and ENT2 have been shown to form dimers^46^, and allosteric modification of inhibitor binding to ENT1 by high concentrations of adenosine has been reported^47,48^. Likewise, sigmoidal transport kinetics have been described for L-adenosine uptake by chromaffin cells^49^. Similar positive cooperativity in substrate-transporter interactions has been noted for other solute carriers such as the citrate transporter SLC13A5^50^.

A goal of this study was to determine if the increased metabolic stability of 2-chloroadenosine would allow more effective analysis of hENT4 nucleoside transport activity. While 2-chloroadenosine is not metabolized by adenosine deaminase, it is still susceptible to phosphorylation by adenosine kinase^20^. This is not an issue when 2-chloroadenosine is used as a substrate for studies of other nucleoside transporters such as ENT1 with uptake time courses on the order of seconds^22,30^, but the slower kinetics of hENT4 raises the possibility that adenosine kinase activity may confound transporter kinetic analysis even when using more stable substrates such as 2-chloroadenosine. This was confirmed by conducting studies in the presence and absence of the cell permeable adenosine kinase inhibitor ABT-702. The inclusion of ABT-702 decreased the maximum accumulation of [^3^H] (prevents the intracellular trapping of [^3^H] as 2-chloroadenosine phosphates), but did not affect the initial rate of [^3^H]2-chloroadenosine influx, (indicating that ABT-702 was not directly inhibiting the transporter). Therefore, in the presence of adenosine kinase inhibitors such as ABT-702, [^3^H]2-chloroadenosine is a suitable substrate for assessing the kinetics of nucleoside transport by hENT4.

As noted by others, D22^34^ and TC-T6000^17^, can inhibit hENT4-mediated substrate influx. The affinity of D22 for blocking 2-chloroadenosine uptake by hENT4 in *SLC29A4*-transfected PK15-NTD cells was consistent with previous studies where 10 μM D22 was able to completely prevent adenosine uptake by hENT4 in *SLC29A4*-transfected MDCK cells^18^ and *Xenopus* oocytes^3^. The ENT1 inhibitors nitrobenzylthioinosine (NBMPR), dilazep, and dipyridamole, which have we have previously shown to have K_i_ values of 2 nM, 10 nM, and 111 nM, respectively, for inhibition of human ENT1 expressed in PK15-NTD cells^25^, were relatively weak inhibitors of hENT4 as expressed in this model. We also show that the lidoflazine analogues soluflazine and draflazine, which are potent ENT1 inhibitors in other systems^28^, are likewise relatively ineffective at inhibiting hENT4. The dipyridamole analogue, TC-T6000, which has been proposed as a more selective hENT4 inhibitor than dipyridamole^17^ had an IC_50_ of 2.3 µM for inhibiting 2-chloroadenosine uptake by hENT4 in *SLC29A4*-transfected PK15-NTD cells. This is more than 30-fold higher than that reported previously for its inhibition of [^3^H]adenosine uptake by hENT4 (74 nM), suggesting that TC-T6000 affinity for hENT4 may be substrate or model dependent. Interestingly, MPP^+^, an established substrate for hENT4^34^, appeared to inhibit only the acidic pH-dependent component of [^3^H]2-chloroadenosine uptake (compared with D22 and TC-T6000 which also inhibited the uptake at neutral pH). This difference may be related to the fact that MPP^+^ is a substrate for the transporter whereas D22 and TC-T6000 are not.

A complication in studying hENT4 activity in endogenous cell models is that it is often co-expressed with other ENT subtypes (specifically ENT1 and ENT2). ENT1 can be potently inhibited with NBMPR, but ENT2 is less sensitive to inhibition. The best inhibitor for ENT2, dipyridamole, inhibits hENT4 to some degree at the concentrations used to maximally inhibit ENT2 (>1 μM). Dipyridamole has also been shown to have non-selective actions on other systems including affecting cAMP and cGMP metabolism^51,52^. Furthermore, the influence of the sodium-dependent concentrative nucleoside transporters (CNTs) in heterogeneous systems is often negated by conducting nucleoside flux studies in the absence of sodium. This is problematic in the study of hENT4, as hENT4 activity has been shown to be sensitive to changes in membrane potential^18,53^. Therefore, we investigated if the pyrimidine nucleoside uridine can be used as an inhibitor for other ENTs and CNTs without affecting hENT4. We showed that 3 mM uridine, which is sufficient to completely block ENT1 and ENT2^54^, and CNT2 and CNT3^33,55^, does not affect hENT4 function. This means that 3 mM uridine can be used alone or in conjunction with NBMPR as an alternative to dipyridamole to suppress ENT1 and ENT2, the main nucleoside transporters in most endogenous cell lines, as well as CNT-mediated transport activity, for study of hENT4.

Other SLC29A family members have been shown to be bidirectional with respect to substrate flux, mediating the flow of substrate across the plasma membrane in either direction down the substrate concentration gradient^35–38^. Under conditions of cellular stress, such as that which occurs in ischaemia-reperfusion events, adenosine can be generated intracellularly from the breakdown of adenine nucleotides and released from cells via equilibrative nucleoside transporters^15,16^. This loss of intracellular adenosine contributes to cellular dysfunction upon reperfusion by limiting restoration of cellular adenine nucleotide pools. It has been shown previously that hENT4 is able to mediate efflux of MPP^+^ ^34^, but the dependence of efflux on pH was not assessed, nor were other substrates besides MPP^+^ tested. It is not feasible to study the efflux of preloaded adenosine as it is rapidly metabolized intracellularly into impermeable adenine nucleotides. However, the finding that PK15-hENT4 cells can accumulate 2-chloroadenosine in a saturable manner upon blockade of its metabolism by adenosine kinase with ABT-702, enabled analysis of [^3^H]2-chloroadenosine efflux by hENT4. Our results show that hENT4 is indeed able to mediate 2-chloroadenosine efflux, and that this efflux could be inhibited by the hENT4 blockers D22 and TC-T6000. Like hENT4-mediated influx, 2-chloroadenosine efflux was enhanced by acidification of the media. The acidic pH-dependent component of hENT4-mediated nucleoside flux does not appear to be proton gradient driven, as we show that collapse of the proton gradient with nigericin did not eliminate the effect of acidic pH on 2-chloroadenosine uptake. Furthermore, we established that an artificially generated proton gradient dissipated within 5 minutes in this cell model, and all of the kinetic influx data shown in Fig. 5 was derived using a 5 minute time point to represent initial rates. Therefore, the acid effect may be due to protonation of amino acid residues of hENT4 affecting transporter function. Alternatively, it may be the protonation of the substrate itself that leads to an enhancement of hENT4-mediated uptake, as has been described for the organic anion transporter OCT2^56,57^. Interestingly, a recent study on the molecular determinants of the pH sensitivity of another SLC29A family member, ENT3, showed that specific amino acids on the face of ENT3 exposed to the acidic environment were important for its acidic pH-dependence^58^. An analogous mechanism may be operating for hENT4.

In summary, our data show that, like ENT1 and ENT2, hENT4 is a bi-directional transporter with respect to 2-chloroadenosine (and presumably adenosine) flux. In addition, we have delineated a positively cooperative relationship for 2-chloroadenosine-ENT4 transporter interactions at high substrate concentrations at acidic pH. We also show that hENT4, in an acidic pH environment, transports 2-chloroadenosine with high affinity at concentrations reflective of the (patho)physiological levels of adenosine These findings suggest that hENT4 may be a novel drug target for the development of therapies aimed at pathologies that generate an acidic environment where endogenous adenosine has been shown to be protective, such as ischaemia-reperfusion injury.

## Supplementary information

Supplemental figure

## Data Availability

All data generated or analysed during this study are included in this published article.
