# Supplementary material for: Bidirectional transport of 2-chloroadenosine by equilibrative nucleoside transporter 4 (hENT4): Evidence for allosteric kinetics at acidic pH
Source: Sci Rep. 2019 Sep 19;9:13555. doi: 10.1038/s41598-019-49929-w (PMC6753126; doi:10.1038/s41598-019-49929-w)

Bidirectional transport of 2-chloroadenosine by equilibrative nucleoside transporter 4 (hENT4): Evidence for allosteric kinetics at acidic pH

David Tandio, Gonzalo Vilas, James R Hammond

Department of Pharmacology, University of Alberta, Edmonton, Alberta, Canada

SUPPLEMENTAL DATA

Supplemental Figure 1: Full size gels from which the cropped images shown in Figure 1 were derived. Unlabelled lanes were samples run on the same gels but from other sources not relevant to the study described in this paper.

A. PCR

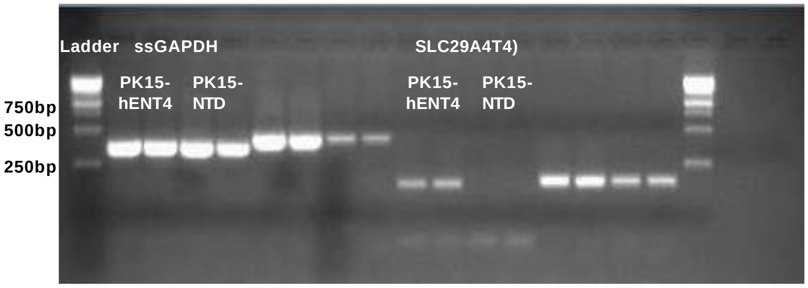

B. Myc Immunoblot

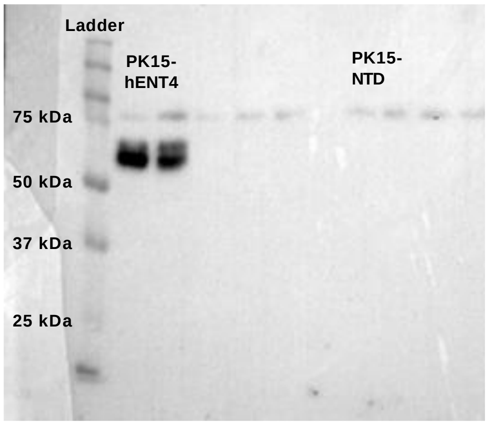

C. GAPDH Immunoblot

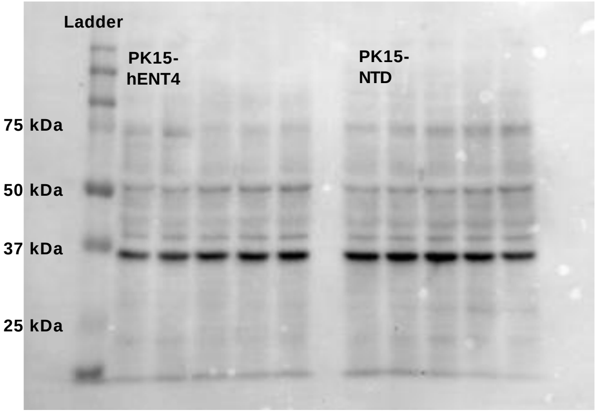

Supplement: Supplementary file 1 — Supplemental figure [file 41598_2019_49929_MOESM1_ESM.pdf]
